# Carcinosarcoma of the Nasal Septum: A Case Report and Review of the Literature

**DOI:** 10.7759/cureus.18546

**Published:** 2021-10-06

**Authors:** Cathleen C Kuo, Francesca Viola, Jason C DeGiovanni, Sam DeVictor, William J Belles

**Affiliations:** 1 Medicine, Jacobs School of Medicine and Biomedical Sciences, University at Buffalo, Buffalo, USA; 2 Otolaryngology, Jacobs School of Medicine and Biomedical Sciences, University at Buffalo, Buffalo, USA

**Keywords:** endoscopy, nasal septum, epistaxis, spindle cell carcinoma, carcinosarcoma

## Abstract

We report a case of a 62-year-old man with epistaxis and right-sided nasal obstruction. Nasal endoscopy revealed an exophytic mass arising from the anterior septum that extended posteriorly to the osteo-meatal complex. Excision with endoscopic sinus surgery was performed. Carcinosarcoma was diagnosed based on histopathology and immunohistochemical studies. The patient declined surgery and opted for chemoradiation therapy for the residual tumor. Six weeks after completion of the treatment, clinical resolution of the right nasal mass was noted. Carcinosarcomas are rare and rapidly growing tumors that have a high recurrence rate and are associated with poor patient prognosis. This report emphasizes the need for patients with prolonged nasal obstruction and epistaxis to consult otolaryngologists and undergo nasal endoscopy for definitive diagnosis and appropriate treatment.

## Introduction

Carcinosarcoma is a highly malignant neoplasm consisting of both epithelial (squamous and columnar) and mesenchymal (fibroblasts, bone, smooth muscle, and cartilage) elements. Histologically, carcinosarcoma has been classified as part of the sarcomatoid carcinoma spectrum, most of which occur in elderly patients (sixth to seventh decade) with a slight male predominance. Other risk factors include a history of smoking tobacco, alcohol consumption, and radiation exposure [1]. In the head and neck region, carcinosarcomas are most commonly found in the oral cavity (63.0%), larynx (17.5%), oropharynx/hypopharynx (11.7%), and esophagus (2.8%) [2]. Extremely rare cases in the nasal cavity and paranasal sinuses have also been described in the medical literature. However, due to the rarity of such reports, the information related to its clinical course, prognosis, and response to treatment are very limited. Herein, we present a rare case of carcinosarcoma arising from the nasal septum.

## Case presentation

A 62-year-old man with a history of hypertension and chronic rhinosinusitis presented to our clinic with complaints of subacute onset right-sided nasal obstruction. He first noticed his symptoms six months ago; however, he noted a rapidly growing mass in his right nostril over the past two months which was causing his symptoms to become more bothersome. Apart from nasal obstruction, he noted recurrent right-sided epistaxis as well as a mass on the right side of his neck that had gotten progressively larger.

**Figure 1:**
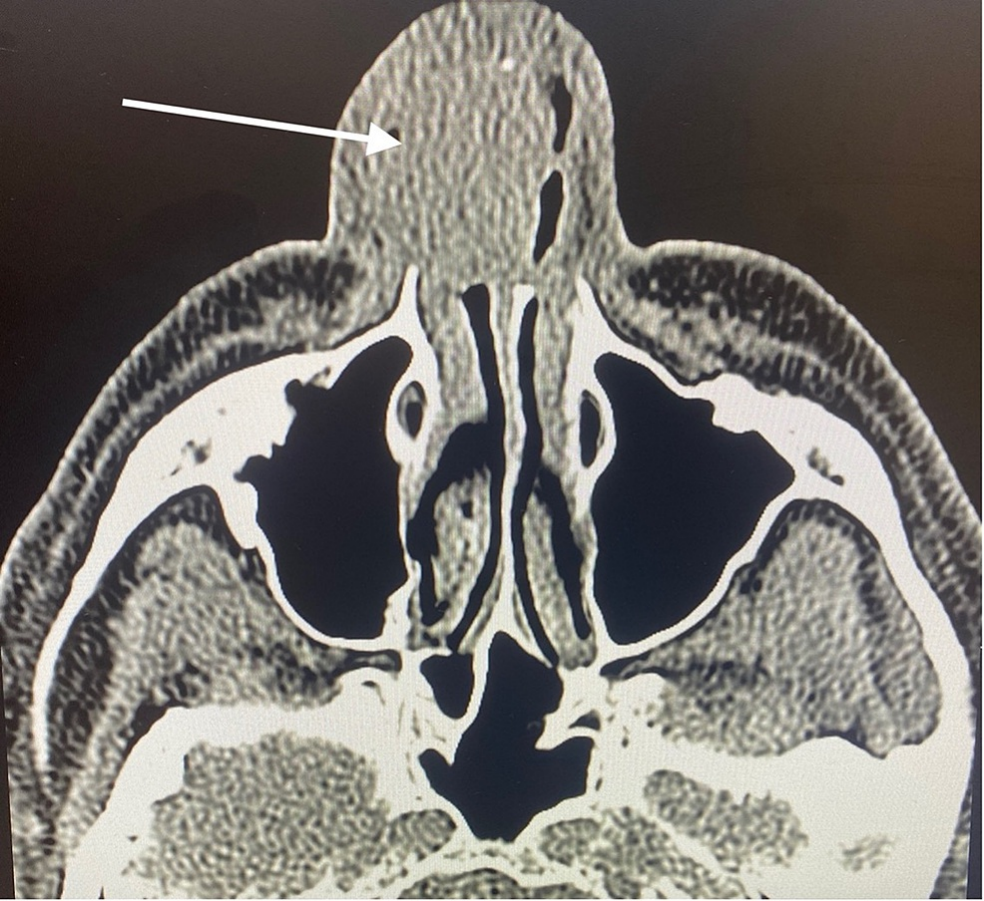
Axial CT scan without contrast at the level of the maxillary sinus in soft tissue window depicting the erosive, right sided nasal mass arising from the cartilaginous septum (arrow).

On exam, he had notable facial asymmetry with the fullness of his right malar region and a mass emanating from the nasal vestibule completely obstructing the right nasal cavity. Nasal endoscopy of the right nostril demonstrated an exophytic, fleshy mass arising from the anterior septum that extended posteriorly causing obstruction of the osteo-meatal complex (OMC). Endoscopy on the contralateral nostril demonstrated leftward deviation of the septum from the mass effect of the lesion. Examination of his neck revealed a firm, fixed, 2 x 1.5 cm mass in level IIB. Computed tomography (CT) imaging of the face and sinuses demonstrated an erosive mass originating from the right cartilaginous septum with erosion and obstruction of the right OMC (Figure 1) as well as acute appearing right-sided ethmoid and maxillary disease. On both physical examination and imaging, there was no ostensible extension to or involvement of other intranasal structures other than the septum.

**Figure 2:**
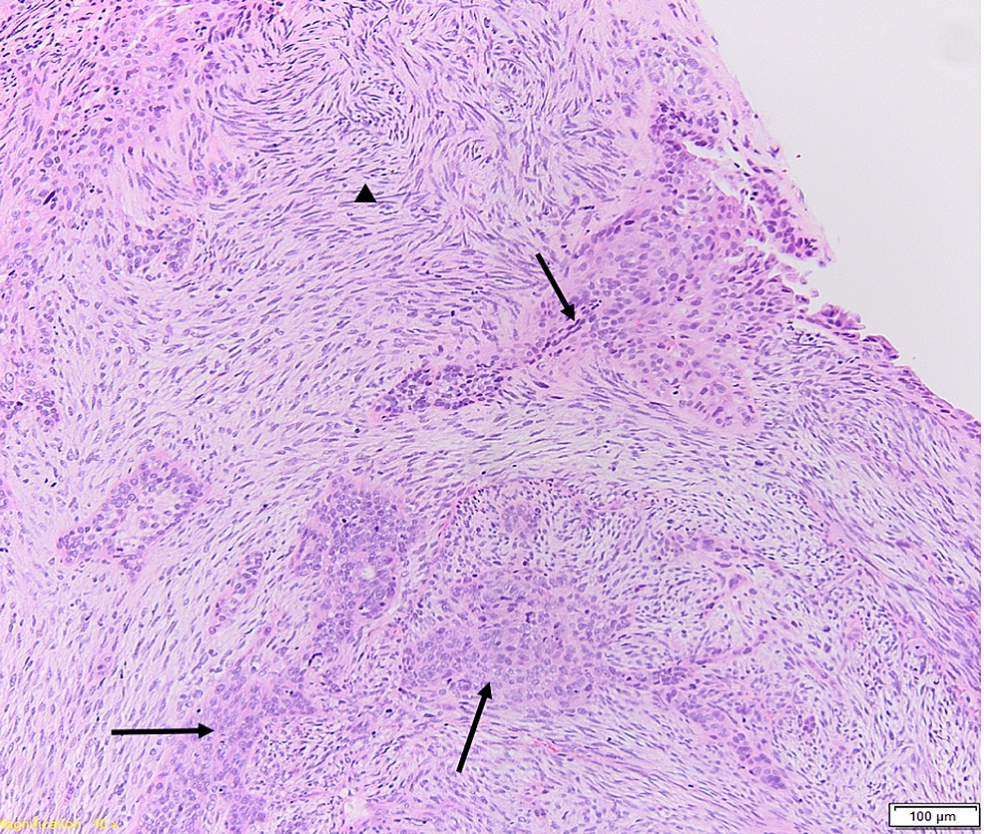
Hematoxylin and eosin (H+E) stain of tissue specimen at 10x magnification demonstrating epithelial (arrows) and sarcomatous components (arrowhead).

The patient was ultimately taken to the operating room for nasal endoscopy and excision of the right-sided nasal mass. Intra-operatively, a 5 x 4 cm mass as previously described was visualized which originated from the anterior and midportion of the septum. Due to an inability to access the posterior margin of the lesion, excision was only feasible through the contralateral nostril. A left-sided hemi-transfixion incision was made and a mucoperichondrial flap was extended backward past the bony-cartilaginous junction. The mass was noted to have eroded through the septum but was not involving the vomer or perpendicular plate of the ethmoid. After disarticulating the bony-cartilaginous junction and freeing the septum from pre-maxillary crest the lesion was removed in its entirety from the right nasal cavity. Pathologic examination of the specimen demonstrated a lesion with both epithelial and sarcomatous components (Figure 2) which stained positively for cytokeratin 5/6 (CK 5/6), Cam 5.2, P40, and epithelial membrane antigen (EMA). The final pathologic diagnosis was carcinosarcoma. The patient opted against an elective neck dissection and instead chose to pursue chemoradiation therapy for the right-sided neck mass.

Ultimately, he completed three cycles of radiation (70 Gy/35 fractions) with concurrent high dose Cisplatin (100mg/m^2^) therapy. Due to underlying kidney function, his third dose of chemotherapy was reduced to 40mg/m^2^. Six weeks following completion of chemoradiation therapy the patient was seen in the clinic and noted to have clinical resolution of the right-sided nasal mass. Apart from some mild xerostomia, the patient denied any symptoms of nasal obstruction or dysphagia. He was scheduled to have a repeat PET scan in four weeks to re-evaluate for signs of residual disease or recurrence.

## Discussion

Carcinosarcoma is a biphasic variant of sarcomatoid carcinoma that can affect any squamous epithelium-lined area of the body, such as skin, genitals, gastrointestinal tract, and respiratory tract. For years, the true pathophysiology of this malignancy was debated and led to a wide variety of diagnostic terms, including spindle-cell carcinoma, pseudosarcoma, pseudosarcomatous carcinoma, metaplastic carcinoma, and polypoid carcinoma [3]. Two antithetical hypotheses regarding the histogenesis of carcinosarcoma have been proposed. The multiclonal theory suggests that a heterogenous tumor arises independently from two or more progenitor cells; however, there is increasing evidence demonstrating that carcinosarcoma originates from a single totipotential stem cell and then differentiates into histologically recognizable epithelial and mesenchymal elements [4]. This distinct combination of tissue components and the low incidence of carcinosarcoma often lead to a misdiagnosis of spindle-cell sarcoma, inflammatory myofibroblastic tumor, fibrosarcoma, malignant melanoma, nodular fasciitis, and synovial sarcoma [4,5].

No systematic review has been done on carcinosarcoma confined solely to the nasal cavity to date. As such, we reviewed the English-language literature in OVID MEDLINE and PubMed with the following keywords: “carcinosarcoma” AND (“nasal cavity” OR “paranasal sinuses” OR “nasopharynx”). Including the current case, four cases of carcinosarcoma confined solely to the nasal cavity have been reported in the literature (Table 1) [6-8]. There was no specific symptom associated with carcinosarcoma of the nasal cavity, but a history of unilateral nasal obstruction, a mass in the nose, and epistaxis were all common clinical features among the reported cases. Careful examination of these masses often revealed a reddish-purple, friable, and polypoid appearance with areas of necrosis and hemorrhage. 

**Table 1:** A review of literature of carcinosarcoma in the nasal cavity

Author, year	Age (years) / Sex	Treatment	Outcome
Ahluwalia, 1996 [6]	40/M	Surgery + Radiation Therapy	Ipsilateral nodal metastases after 2 months (disease-free following modified radical neck dissection)
Gupta, 2013 [7]	29/M	Surgery	Disease-free after 6 months
Mistry, 2016 [8]	50/F	Surgery	Disease-free after 6 months but complained of watering from left eye due to wide surgical excision
Our case, 2021	62/M	Chemotherapy + Radiation Therapy	Disease-free after 6 weeks

Given the aggressive nature and high rate of recurrence, the management of carcinosarcoma poses a challenge. From the known cases (Table 1), the most common treatment modality was surgery alone (66.6%), followed by surgery with adjuvant radiotherapy (33.3%). In our case, the patient declined surgery and opted for chemotherapy and radiotherapy alone. The largest landmark study using the Surveillance, Epidemiology, and End Results (SEER) database demonstrated that patients with sinonasal carcinosarcoma had a lower recurrence rate when treated with surgical excision followed by adjuvant radiation therapy than treated with surgery alone [9]. However, several studies have reported post-radiation nasal dryness, chondritis as well as necrosis and sloughing of the nasal tip, which may require further plastic reconstruction [10-11]. The paucity of cases to date creates an obstacle to determine the clinical effectiveness of radiotherapy with statistical significance. 

Carcinosarcoma is known to have a grave prognosis, with overall mortality as high as 60% and 42% in 30 months [11]. The overall survival of patients with carcinosarcoma in the nasal cavity (Table 1), nonetheless, was great compared to patients with sinonasal carcinosarcoma. It is worth noting that the patient described by Ahluwalia et al. [6] developed ipsilateral nodal metastasis two months following treatment completion. Despite the metastasis, which almost always leads to an unfavorable outcome, this patient showed no evidence of malignancy after modified radical neck dissection. We postulated that the better surgical outcome may be due to the polypoid growth pattern observed in carcinosarcoma of the nasal cavity, whereas carcinosarcoma of the sinonasal region often presents with aggressive and infiltrating behavior [9]. Moreover, the nasal cavity is more easily accessible for surgical intervention than the sinonasal tract.

## Conclusions

Carcinosarcoma of the nasal cavity is an extremely rare neoplasm with non-specific symptoms. Excisional biopsies are often required to make an accurate diagnosis because of the diversity of tissue components. Based on our analysis of historical data, the primary treatment modality for carcinosarcoma of the nasal cavity is surgical resection. Our study demonstrated the feasibility of chemotherapy and radiation alone in treating carcinosarcoma; however, more studies are required to confirm its effectiveness and elucidate the role of chemotherapy. 
